# Problematic Game Play: The Diagnostic Value of Playing Motives, Passion, and Playing Time in Men

**DOI:** 10.3390/bs5020203

**Published:** 2015-04-30

**Authors:** Julia Kneer, Diana Rieger

**Affiliations:** 1 Erasmus School of History, Culture and Communication (ESHCC), Erasmus University Rotterdam, PO Box 1738, Rotterdam, 3000 DR, The Netherlands; 2 Department of Media and Communication, University of Mannheim, Mannheim 68159, Germany; E-Mail: diana.rieger@uni-mannheim.de

**Keywords:** Internet gaming disorder, problematic game play, obsessive passion, game play motivations, playing time

## Abstract

Internet gaming disorder is currently listed in the DSM—not in order to diagnose such a disorder but to encourage research to investigate this phenomenon. Even whether it is still questionable if Internet Gaming Disorder exists and can be judged as a form of addiction, problematic game play is already very well researched to cause problems in daily life. Approaches trying to predict problematic tendencies in digital game play have mainly focused on playing time as a diagnostic criterion. However, motives to engage in digital game play and obsessive passion for game play have also been found to predict problematic game play but have not yet been investigated together. The present study aims at (1) analyzing if obsessive passion can be distinguished from problematic game play as separate concepts, and (2) testing motives of game play, passion, and playing time for their predictive values for problematic tendencies. We found (*N* = 99 males, Age: *M* = 22.80, *SD* = 3.81) that obsessive passion can be conceptually separated from problematic game play. In addition, the results suggest that compared to solely playing time immersion as playing motive and obsessive passion have added predictive value for problematic game play. The implications focus on broadening the criteria in order to diagnose problematic playing.

## 1. Introduction

Internet gaming disorder, also known as gaming addiction, has come into the focus of public debates and scientific research. This phenomenon seems to be classified as a new disorder having risen in the digital century, especially with the occurrence of online games. Several information centers and treatment possibilities have already existed for years. According to different sources, 3%, 9% or 11% of players can be considered problematic players [1,2,3]. Problematic playing behavior is often defined by having problems with real life due to excessive playing; therefore, problematic players are often considered to be addicts/to have developed an Internet gaming disorder. 

As reaction towards this development, the APA (American Psychiatric Association) decided to list “Internet Gaming Disorder” in Section III of the Diagnostic and Statistical Manual of Mental Disorders 5 (DSM 5) in order to encourage scientists to conduct more research to determine if this disorder should be included in the next DSM generation. Most studies that focused on diagnostic instruments [4] and risk factors [5] could rarely identify addicts but players with problematic playing behavior. Most studies on this topic mostly analyzed subjects that ranked between “no problematic playing behavior” and “behavior”, while only a few studies were able to identify addicted players. We have to keep in mind that despite ambivalent findings, problematic playing behavior and addiction was found to exist and to cause heavily troubles in daily life. These daily life struggles due to problematic playing behavior are reason enough to investigate further risk factors and their combination in order to detect causes and help players to stay healthy. In response to this need to clarify the circumstances of Internet gaming disorder, recent attempts following the inclusion into the DSM have concentrated on achieving a consensus between experts on a cross-cultural level concerning the assessment of Internet gaming disorder [6]. 

It is still an open question why some players develop more problematic game play than others. One idea is that people differ with regard to their *motivation* on why they play and that some motivations may have a stronger impact on problematic tendencies than others. In addition, *passion* for game play might become obsessive, which can result in problematic game play. Since normal playing behavior and problematic playing behavior are located on a continuum [7], there might be motives and a specific form of passion, which contribute more to problematic game play than others. In order to prevent the development of problematic game play, it is important to investigate which motive is prone to be a potential risk factor as well as to investigate the role of passion. The present study therefore aims at testing if known motives for game play and game play passion contribute to problematic game play.

In order to structure the copious literature on gaming addiction/Internet gaming disorder, Kuss and Griffiths [7] presented a systematic review, providing a framework that classifies the existing studies. Based on the empirical literature, they argued that Internet gaming disorder followed a continuum, ranging from antecedents in aetiology and risk factors (1) up to the development of a “full-blown” addiction; (2) to ramifications in terms of negative consequences, and (3) potential treatment. 

With regard to the first category, many risk factors have been identified. In particular, playing time is still considered to be a main diagnostic criteria, as it was found to be strongly connected with negative outcomes of digital game play [8]. But playing time as a single risk factor is not sufficient to explain problematic playing behavior [8]. 

Risk factors have been found among personality traits (e.g., low self-esteem, [9,10]), social settings ([11], e.g., lonliness, [12]), and more recently playing motives [8,13] as passion (e.g., [14]). Personality traits found to contribute to problematic game play can be subsumed under three characteristics; introversion, neuroticism, and impulsivity [7]. According to Kuss and Griffiths [7], personality traits are not exclusive risk factors for problematic game play, but rather general problematic tendencies. Their conclusion is that it is not yet possible to draw upon the aetiological significance of these findings. With regard to playing motives, Kuss and Griffiths [7] identified 13 studies which focused on the motives for playing and problematic game play behavior. Considering that before playing becomes problematic, it can be considered to be a normal hobby for young adults [15], specific game play motivations might play a major role in the development of problematic game play. Overall, Kuss and Griffiths [7] concluded that it was particularly motivations related to dysfunctional coping, socialization, and personal satisfaction that served as risk factors for developing problematic game play. Interviews which were conducted with councilors who work with problematic players confirmed this finding [16].

Indeed, it is a vicious circle since digital games seem to offer perfect coping strategies for all real life problems that have been identified as risk factors for problematic playing behavior. Especially the virtual worlds of Massively Multiplayer Online Role-Playing Games (MMORPGs) offer the opportunity to engage in a virtual life that is completely controlled by the player. Players are able to create their own so-called avatar (virtual character), which corresponds to their ideal identity. New families can be joined and new friends can be found. Players have the opportunity to achieve something, even if real work or school life seem to fail. In addition, online games can be used to cope with real-world related stress in general by offering the opportunity to escape. 

Yee provided a framework which categorizes playing motives to three main components and explain motives for playing especially MMORPGs: achievement, social interaction, and immersion [17]. These three playing motives provide an idea about why something starts to play and stays with this hobby. Within these playing motives, gender differences were found to occur: While men are more driven by achievement motivations, women turn to gaming due to social interaction [18]. Further research on the topic of game play motivations applied self-determination theory and was successful to find that playing games can satisfy three intrinsic needs: autonomy (being in control), achievement (feeling competent), and relatedness [19]. If these needs are satisfied, enjoyment will be reached [20]. However, problematic game play might no longer be related to actual enjoyment resulting from media entertainment but to coping with real life problems by playing games. In this case, intrinsic need satisfaction is not the driving force and motivation for game play based on enjoyment changed. Instead of entertainment games now offer an escape strategy from real life troubles what is closely related to the immersion motivation found by Yee [17]. Caplan, Williams and Yee [21] directly tested this assumption and found that immersion playing motivation was the only playing motive that had predictive value in fostering problematic Internet use. 

Indeed, Hellström, Nilsson, Leppert, and Åslund [8] provided evidence for social interaction and achievement to decrease problematic game play while immersion had a positive relation with problematic tendencies [8]. The higher the motive immersion was judged, the more problematic was the playing behavior. The same results were found by Kneer and Glock [13]. Immersion was again found to be the most risky playing motivation to develop problematic playing behavior. In these two studies, playing motives explained problematic playing behavior better than playing time alone. This is first evidence showing that playing motives—especially immersion—can contribute to an understanding of problematic game play. The first goal of our study was to replicate these results by showing that playing motivations have predictive value for problematic game play and to test whether immersion is the most risky motive for the development of problematic game play, while social interaction and achievement motivations are not dangerous. 

Besides motives, the differentiation between healthy and problematic game play might be explained by a further criterion: *passion*. The dualistic model of passion differentiates between harmonious and obsessive passion and is defined by the possibility to control the engagement in an activity [22]. Harmonious passion means that persons accept the activity as part of their identity and consider it as important but are still free to choose if and how they participate. Obsessive passion is given when the activity controls important parts of the identity such as self-esteem or social acceptance and/or the person depending on the excitement resulting out of the activity. In other words: under harmonious passion, the person controls the activity, while under obsessive passion, the person is controlled by the activity. Both aspects of passion and their interaction with problematic game play were investigated [23]. Utz, Jonas, and Tonkens [14] introduced the concept of harmonious and obsessive passion to distinguish between modes of game play, with obsessive passion forming a rather susceptible factor to contribute to problematic playing behavior. In line with this, Lehenbauer-Baum and Fohringer differentiate between addicted players and highly engaged players and conclude that cognitive salience, tolerance, and euphoria, which can be associated with harmonious passion for an activity, are not suitable to diagnose Internet gaming disorder [24]. Other criteria, such as tapping interpersonal conflicts, withdrawal symptoms, relapse and reinstatement, and behavioral salience that can be related to a lack of control and thereby obsessive passion, were related to addictive behaviors. 

However, at this point, it is unclear if obsessive passion can be really differentiated from problematic game play. So far, no study clearly distinguishes both concepts or were any results in terms of discriminant validity given. Especially discriminant validity has to be taken into account if a new predictor is presented [25]. Without this analysis it remains unclear if obsessive passion is measuring the same underlying concept as problematic game play. Thus, our study’s second goal aimed at testing obsessive passion against problematic game play as different concepts. 

Lemmens *et al.* [12] describes immersion, or involvement, to be one aspect of problematic gaming, as well as obsessive passion. Although immersion as a playing motive and obsessive passion were both found to be connected to problematic game play in previous studies, they were not investigated together. Thus, the third goal of our study was to combine playing motives, passion, and playing time and to investigate their role in problematic game play. 

Based on previous research, we expect that: (H1) obsessive passion can be distinguished from problematic game play based on discriminant validity analysis; (H2) playing time has only predictive value for problematic game play if no further predictor is taken into account; (H3) immersion as playing motive is a significant predictor for problematic game play; (H4) obsessive passion has predictive value for problematic game play; (H5) social interaction as well as achievement as motives cannot predict problematic game play; and (H6) harmonious passion has no predictive value for problematic game play.

## 2. Method

### 2.1. Participants and Design

Our predictors for the problematic game play score were social interaction, achievement, immersion, obsessive passion and harmonious passion concerning digital game play and playing time. To test our hypotheses we needed *N* ≥ 15*Predictor number participants with digital game playing experience [26]. The amount of predictors resulted in *N* ≥ 90. We recruited 99 German players (all male, Age: *M* = 22.80, *SD* = 3.81) by university mailing lists, personal contact, and by attending LAN-parties. Most participants (60.60%) were employed after finishing a second-degree school education (in German: “*Realschulabschluss*”) and had finished work training (in German: “*Geselle*”). All other participants were either still studying (28.30%) or finished their studies and are currently employed (11.10%). Thus, the educational level of our sample is representative for this age group.

They all participated voluntarily and without payment. In order to avoid problems due to uncontrolled online survey settings, we asked participants to come to our laboratory and fill out our questionnaires on the computer. This procedure was the best to count for social desirability, since all questions were shown via computer screen and not asked during personal interviews. Being in a laboratory environment still helped to control for external factors, which might influence studies done via online questionnaires. All participants had digital game playing experience: *M_hours per week_* = 23.41, *SD =* 17.83 *M_years_ =* 10.99, *SD =* 5.31. 

### 2.2. Procedure

We used the six questions to measure game play motives (immersion, achievement, social relatedness), which were tested in a previous study for German players [27]. Participants were asked to judge their playing motives explicitly on a 7-point-Likert-Scale (1 = does not fit at all; 7 = fits perfectly) including two questions for each of the three dimensions: “When I play digital games this is motivated by…” For social interaction these were: (1) Friendship and (2) Joy due to support. For achievement they were: (3) Achievement and (4) Competition. Finally, for immersion they were: (5) Stimulation; and (6) Escapism.

Participants were then required to answer five questions based on the German questionnaire of Grüsser and Thalemann (2006) concerning problematic game play on a 6-point-Likert Scale (1 = does not fit at all; 6 = fits perfectly). This questionnaire was successfully used in recent studies with German players [12] and relates to most criteria that are currently suggested for Internet gaming disorder diagnoses by the APA. (1) “Have you ever missed a meeting with friends or your family because you played digital games instead?” (criteria 7: problems with family and friends due to game play); (2) “Do you neglect your duties due to your playing behavior?” (criteria 9: job or school problems due to gaming; criteria 4: control problems); (3) “Do you think about playing digital games while doing something else?” (criteria 1: preoccupation within thoughts, obsessive thoughts; criteria 5: interest loss in other hobbies); (4) “Have you ever slept less than eight hours due to digital game play?” (criteria 6: continues gaming despite knowing about problems caused by gaming; criteria 3: tolerance, more time needs to be invested); and (5) “Do you feel nervous if you are not able to play any digital games?” (criteria 2: withdrawal problems). 

In order to assess obsessive and harmonious passion for game play, we translated the questions from Vallerandt *et al.* [22] into German. At the end of the experiment participants filled out their answers to demographical questions including playing time per week and playing time [28] per genre (see Table 1).

**Table 1:** Means and SD for playing time per genre in hours per week.

Genre	*M*_hours per week_	*SD*
Action	2.60	4.40
Beat	0.79	2.34
First person shooter	5.07	6.60
Jump ‘n’ run	0.61	1.25
Role-playing	3.49	5.12
Real-time strategy	3.67	4.91
Simulation	1.23	2.74
Sport	0.90	2.60
Miscellaneous	1.70	2.84

## 3. Results

### 3.1. Testing Pre-Assumptions for Regression Analysis

We calculated the total sum for the problematic game play score (Cronbach’s α = 0.613), the obsessive passion score (Cronbach’s α = 0.753), the harmonious passion score (Cronbach’s α = 0.796), the immersion score, the social interaction score, and the achievement score. None of the scores was normally distributed, all *p* < 0.10. Therefore, we log-transformed all scales (Field, 2009). No effects found before log-transforming changed. 

### 3.2. Main Analyses

#### 3.2.1. Distinguishing Problematic Playing Behavior from Obsessive Passion

In order to test our first hypotheses we calculated the discriminant validity concerning problematic playing behavior and obsessive passion. We used the formula from Campell and Fiske (1959):

(1) $$\frac{r_{ij}}{\sqrt{r_{ii}r_{jj}}}$$

With:

*r_ij_* = correlation between problematic playing behavior and obsessive passion (0.505),
*r_ii_* = reliability for problematic playing behavior (0.613), and
*r_jj_* = reliability for obsessive passion (0.753).

The discriminant validity of 0.743 is lower than 0.85 and thus, confirms that both concepts can be distinguished [25], which supports our hypothesis (H1). Obsessive passion will now be used as a predictor for problematic game play. 

#### 3.2.2. Predictive Value of Motives, Passion, and Time

Correlation between the predictor variables were all *r* < 0.80. All 1/VIFs were above 0.20 and Durbin-Watson was 1.74. No pre-assumption for regression analyses was violated by our data. We conducted a hierarchical regression analysis with the log-transformed data. The problematic game play score was the criterion. Playing time was entered in the first block. In the next block, the obsessive passion score and the immersion score were entered. The third and final block included the scores for social interaction, achievement, and the harmonious passion score (see Table 2) for beta weights and values for explained variance). 

**Table 2:** Standardized beta weights and *R*^2^ of the hierarchical regression analyses with ratings on the game addiction scale as criterion.

Title	Model 1	Model 2	Model 3
Predictor			
playing time	0.22 *	0.01	−0.01
obsessive passion		0.44 ***	0.42 ***
immersion		0.24 **	0.26 **
harmonious passion			0.07
social interaction			−0.12
achievement			0.17
	*R*^2^_adj_ = 0.04 **	∆*R*^2^ = 0.29 **	∆*R*^2^ = 0.03 **
	*p* < 0.05	*p* < 0.001	*n.s.*

Note: *p < 0.05, **p < 0.01, **p < 0.001.

When playing time was used as single predictor, only 2.2% of variance was explained. As expected (H3 and H4), the second block showed that immersion together with obsessive passion explained the most part of the variance in problematic game play while playing time had no further predictive value (H2). These two factors together explained 29% of the variance in problematic game play, which is rather high. Social interaction, achievement, and harmonious passion had no further significant predictive value (H5 and H6). 

#### 3.2.3. Correlations between Game Genres, Problematic Game Play, Passion, and Playing Motives

In order to test if motivations for game play and problematic game play behavior differ between different game genres, we calculated correlations between those scales. None of the correlations between hours spent per week for different game genres and the problematic game play score reached significance. For obsessive passion we found significant correlations for hours spent per week for action, *r* = 0.23, *p* < 0.05, for beat, *r* = 0.37, *p* < 0.001, and role-playing, *r* = 0.20, *p* < 0.05. Harmonious passion correlated significant with beat, *r* = 0.31, *p* < 0.005, and miscellaneous, *r* = 0.20, *p* < 0.05. Social interaction as playing motive did not correlate significant with time spent on different game genres while achievement was found to correlate significant with first person shooter, *r* = 0.20, *p* < 0.05, and immersion with beat, *r* = 0.26, *p* < 0.05.

## 4. Discussion

Our study focused on how digital game playing motives and passion for game play are associated with problematic game play behavior and whether they are better predictors for problematic tendencies than solely playing time. In terms of playing motives, Hellström *et al.* [8], as well as Kneer and Glock [13] found immersion to be the most important risk factor for problematic game play. We could replicate these findings and found immersion as the only motive thath had predictive value for problematic game play (goal 1). Social interaction and achievement were not found to be significant predictors. Therefore, we conclude that immersion as playing motive differs from social interaction and achievement. Immersion seems to be related to wrong coping strategies while social interaction and achievement might contribute to well-being instead of increasing problematic game play. 

Some might criticize that playing motivations are also just a result of underlying daily life problems. Especially immersion might be the outcome of heavy troubles resulting in the wish to escape all problems by playing games. Diagnostic instruments all deal with difficulties regarding the tendency to answer social desirable and/or reactant towards daily life problem questions. In this case, dishonest answers are given in order to avoid criticism and feelings of shame and guilt. Including questions about playing motivations might be a way out of this dilemma. Our results suggest that especially immersion is linked to problematic game play. Thus, immersion questions could be used as possible indicator of problematic game play. 

Concerning passion, we first analyzed if obsessive passion can be conceptual distinguished from problematic game play. Obsessive passion is defined as “being controlled by the activity” what is also included in most definitions of addictive behaviors. Thus, it remained unclear if obsessive passion is measuring the same underlying concept than problematic game play. Our results confirmed that obsessive passion and problematic game play can be distinguished on a conceptual basis based on their discriminant validity (goal 2). Thus, obsessive passion as measurement for being under control of the activity does not automatically point to real life problems. Still, obsessive passion can be of predictive value in terms of the development of problematic game play.

Wang *et al.* [23] found that obsessive passion, in contrast to harmonious passion, is connected to problematic game play [23]. Our results were in line with these findings. Obsessive passion was found to have predictive value for problematic game play, while harmonious passion was unconnected. Being obsessed by games results in loss of control, which is a typical indicator of problematic behavior. In contrast, harmonious game play is not unhealthy at all. 

In line with Kneer and Glock [13], we did only find a predictive value of playing time if playing time was analyzed as single predictor. In addition, the predictive value of the first regression model was low. Taking into consideration that playing time is still very often used as a main criterion in diagnosing problematic game play, while passion and playing motives are often not included in diagnostic instruments, our results might be a contribution for future diagnostic instruments and intervention programs. The importance of immersion as a playing motive and the clarification whether playing passion is obsessive seem to have better diagnostic value than playing time alone (goal 3).

Analyzing problematic game play and the role of motivation and passion for different game genres we found that problematic game play was not related to one specific game genre. This result is not in line with findings from other studies, which found especially online role-playing games to correlate with addictive tendencies [21]. This might be due to two reasons. First, our specific sample spent most time with fist person shooters, and not with online role-playing games. Thus, online role-playing games were not the favorite game genre of this sample. Second, our sample did not include any addictive players. In case of addiction, this correlation might turn out to be significant again. 

Our study is limited in several ways. We only assessed playing motives, obsessive and harmonious passion scores, playing time, and the self-reported problematic game play scale once. Future studies should investigate whether immersion as the main playing motive combined with obsessive passion for game play leads to the development of problematic game play over time. In addition, as other studies on this topic we had no addicted players in our sample. Upcoming research should focus on addicted players in order to investigate their motives and obsessive playing scores compared to healthy players.

The use of the questionnaire for problematic game play is as well critical. One criterion of the nine suggested of the APA was not linked to a question at all, which addresses negative feelings (e.g., guilt, helpless) due to game play. We still decided to use this measure due to its successful application in a past German study on problematic game play [12]. However, there are new developed measurements for problematic game play [6], which should be used in future studies [29]. Our sampling strategy was not randomized but convenient. We decided for this sampling strategy to reduce socially desirable answers or reactance bias by inviting players into our laboratory. After all, studies that address problematic game play include questions that might cause shame and guilt within players, which might cause participants to lie about their behavior if asked online. Our method to invite players gave them more control feelings, which reduces socially desirable answers and reactance. Compared to online studies, we could reduce problematic answers but still had the problem of self-selection. This of course has impact on the generalizations of our results as self-selection for psychological studies always lack probability sampling. Further studies should aim for a random sampling strategy in order to have results that are more generalizable. In addition, our sample was rather small and our study only included male participants. Due to the high costs of conducting a study in a laboratory, we fist calculated the minimum number of participants we needed to analyze our main hypotheses (see section Participants and Procedure). The reason for only including male participants was that only four female participants agreed to take part in our study. Due to this low number, we decided to only include male participants. However, future studies should include bigger samples and include female players as well.

## 5. Conclusions

In this study, game play motives, passion for game play as well as playing time were analyzed as predictors for problematic game play. Our results showed that immersion as playing motive and obsessive passion for game play have significant predictive value for problematic game play while playing time had only significant influence on problematic game play if used as single predictor. Concerning the development of future diagnostic instruments, game play motives and passion should be discussed as criteria.
